# Structural and Mechanistic Features of ClyA-Like α-Pore-Forming Toxins

**DOI:** 10.3390/toxins10090343

**Published:** 2018-08-23

**Authors:** Bastian Bräuning, Michael Groll

**Affiliations:** Center for Integrated Protein Science Munich (CIPSM), Department of Chemistry, Technische Universität München, Lichtenbergstrasse 4, 85747 Garching, Germany

**Keywords:** pore-forming toxins (PFT), virulence factors, structural biology, x-ray crystallography, cryo-electron microscopy

## Abstract

Recent technological advances have seen increasing numbers of complex structures from diverse pore-forming toxins (PFT). The ClyA family of α-PFTs comprises a broad variety of assemblies including single-, two- and three-component toxin systems. With crystal structures available for soluble subunits of all major groups in this extended protein family, efforts now focus on obtaining molecular insights into physiological pore formation. This review provides an up-to-date discussion on common and divergent structural and functional traits that distinguish the various ClyA family PFTs. Open questions of this research topic are outlined and discussed.

## 1. Introduction

Pore-forming toxins (PFTs) of pathogenic bacteria are well-characterized virulence factors. They belong to an ancient and largely diverse protein family. PFTs are found across Gram-negative and -positive clades of bacteria, with members amongst human, insect and plant pathogens [1,2]. Recent progress in the structural interrogation of increasingly complex pore-forming mechanisms has been fueled by the advances in cryo-electron microscopy (cryo-EM) [3,4,5,6,7,8], allowing the structural determination of heterogeneous pore assemblies. Together with X-ray crystallography of the soluble toxin components, we now have a good grasp of how different PFTs achieve the transition to membrane-bound oligomers. 

Depending on the secondary structural nature of the membrane perforating channel, PFTs are divided into two families: α-PFTs form α-helical pores, while β-PFTs produce β-barrel pores. Historically, β-PFT structures have been overrepresented in the protein data bank (PDB), while reports on α-PFTs are only recently becoming more frequent [6,7,8,9,10]. A comprehensive overview of known bacterial PFT architectures is reported in a review by Da Peraro and Van Der Goot [2]. 

## 2. The Common Structural Topology of ClyA-Like Toxin Components

**Figure 1:**
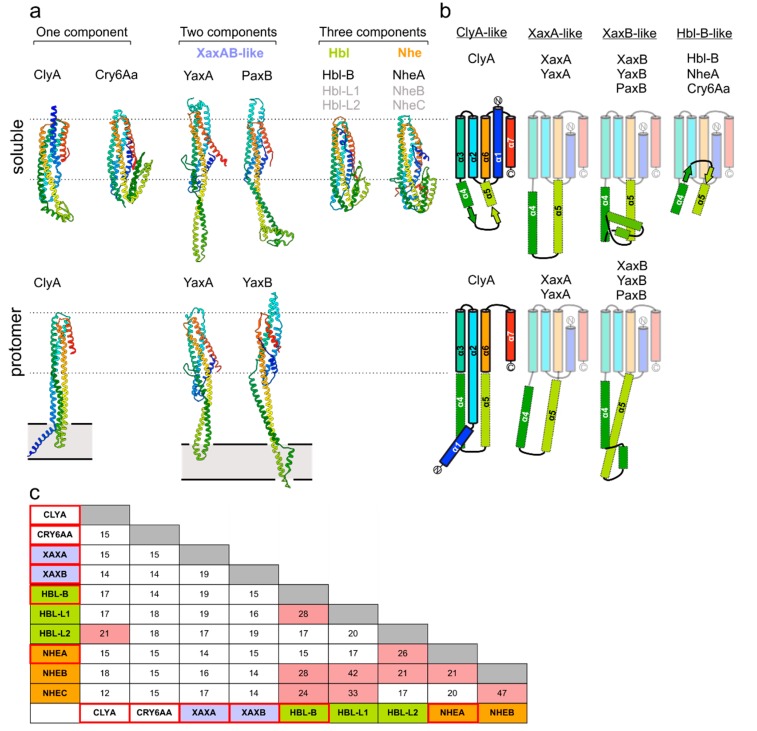
Overview of ClyA-like toxin structures. (**a**) Cartoon depiction of soluble (top) and protomeric (bottom) states of ClyA family member structures reported so far. PDB ID: 1QOY (soluble ClyA) [17], 2WCD (protomeric ClyA) [9], 6EK7 (soluble YaxA) [7], 6EL1 (protomeric YaxA, protomeric YaxB) [7], 6EK4 (soluble PaxB) [7], 4K1P (soluble NheA) [15], 5KUC (Cry6AA) [11], 2NRJ (Hbl-B) [14]. Ribbons are colored blue (N-terminus) to red (C-terminus). Dashed lines highlight the 5HB core motif and grey rectangles delineate the approximate membrane boundaries. (**b**) Topology diagrams of ClyA family members in soluble and protomeric states. (**c**) Comparison of pairwise amino acid sequence identities (%). Proteins with available crystal structures are framed in red. Pairs with a sequence identity above 20% are highlighted with a pink background. Alignments were performed with Clustal Omega [18].

Crystal structures are currently available for at least one type of soluble subunit from each known and compositionally diverse group within the ClyA toxin family (Figure 1a): ClyA [9] and Cry6Aa [11] represent one-component toxins; members of the XaxAB family contain two modules [7,8,12,13] and the Nhe and Hbl systems from *Bacillus cereus* are assembled from three distinct building blocks [14,15]. A common structural frame, harboring a five-helix bundle motif (5HB; α1, α2, α3, α6 and α7), is a general feature present in all toxin components analyzed to date (Figure 1b). However, the toxin groups differ in the length and structure of the connecting region between the α4 and α5 helices, which include experimentally validated membrane-active moieties (see below). In addition, bioinformatics and biochemical studies have mapped putative parts of the lytic apparatus to corresponding protein regions in Nhe and Hbl [15,16], whose peculiarities still need to be verified. Despite the conserved topology of the 5HB fold, members within and between each of the wider ClyA family share low sequence similarity, ranging between 15–45% (Figure 1c). Most probably, this fact resulted from the divergent evolution of the members of this family of toxins, providing them with specific mechanistic singularities in achieving the lytic pore arrangements. Nevertheless, as outlined in the following sections, the common 5HB partakes in forming important intersubunit contact sites.

## 3. Homooligomeric ClyA and Cry6Aa Toxins

The first reported structure of an α-PFT was the homododecameric ClyA pore complex, which was solved by X-ray crystallography in 2008 [9]. The mechanism of ClyA pore formation has been well described [19,20]. More recently, Cry6Aa was discovered as a new member of the structurally diverse group of the so-called ‘crystal proteins’ from *Bacillus thuringiensis*. These PFTs have gained widespread biotechnological attention as orally active insecticidal and nematocidal toxins [21,22,23]. Crystal structures of Cry6Aa confirmed that the protein belonged to the wider family of ClyA-like proteins [11], which are topologically closest to Hbl-B and NheA (Figure 1b). Current evidence points to a homooligomeric assembly state of Cry6Aa, and the pore formation has recently been verified [24]. It is curious that within the ClyA family, Cry6Aa holds greatest structural resemblance to Hbl-B and NheA (see Figure 1a), which are components of the tripartite Hbl and Nhe toxins, respectively (see below). Thus, the assembly mechanism most likely differs between the two known one-component systems in this toxin family.

## 4. Two-Component Heterooligomeric ClyA-Like Toxins

**Figure 2:**
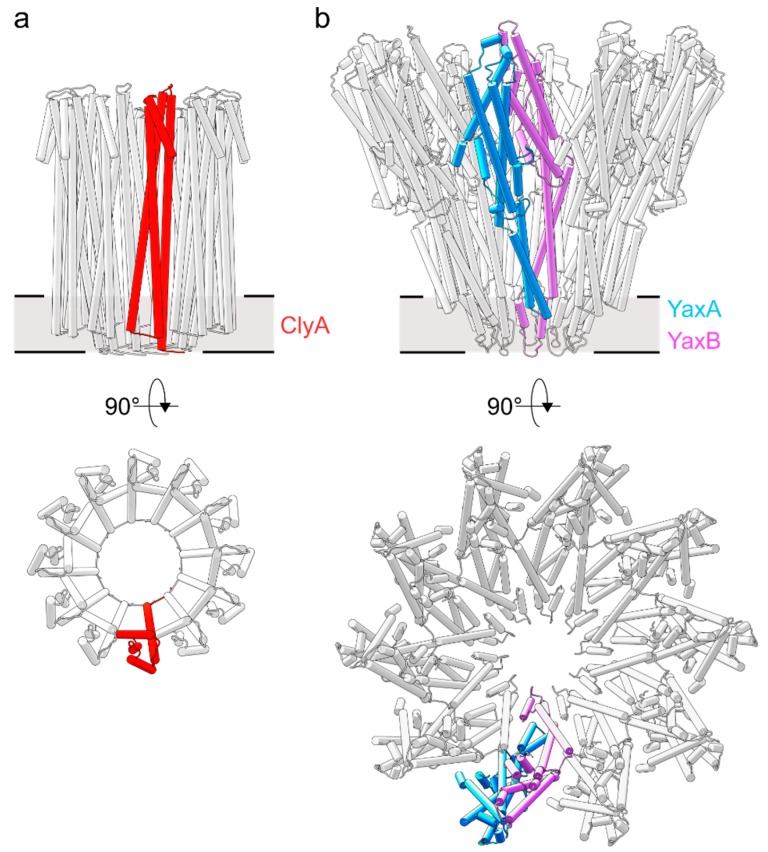
Structural comparison of ClyA and YaxAB pore architectures. (**a**) The homo-12meric ClyA toxin (PDB ID: 2WCD) [9]. The protomeric unit is colored red. (**b**) The hetero-20meric YaxAB toxin [7] (PDB ID: 6EL1). The protomeric subunits of the YaxA-YaxB heterodimer are shown in blue and pink, respectively.

Currently, cryo-EM reconstructions of two orthologous XaxAB-like pores from *Xenorhabdus nematophila* (XaxAB) [8] and *Yersinia enterocolitica* (YaxAB) [7] have been reported. These structural characterizations allow for direct comparison of pore architectures within the wider family of ClyA-like toxins (Figure 2). While limited heterogeneity in ClyA pores can be observed [25], XaxAB-like toxins possess broad distributions, ranging from 8–16-fold symmetric pores [7,8]. Despite apparent differences in stoichiometry, the assembly principle of the two toxin components in XaxAB and YaxAB is uniform. The central core structure is composed of a tightly interacting A-B pair, representing the quasi-protomeric unit, which interacts with adjacent heterodimers in *trans*. Here, a large interface is buried between the 5HB motifs within each A-B dimer, whereas the extended coiled-coils are solvent exposed. This latter observation contrasts with the ClyA homooligomer, wherein extensive intermolecular contacts are formed along the entire protomer. The interface between A and B protomers is resumed at the membrane-active domains formed between the protein regions encompassing helices α4 to α5. Notably, as previous crystallographic studies have shown [9], the soluble monomer of ClyA undergoes significant conformational changes to form the transmembrane pore, which in particular entails a large rearrangement of the helix α1 (Figure 1a,b). In the XaxAB-type system, the 5HB integrity is conserved following the conformational transition to the membrane-bound protomer (Figure 1a,b). As identified through recent biophysical work on ClyA [19], the major structural changes undergone by this one-component PFT are accompanied by a transiently populated, off-pathway intermediate with a molten globule character. Why toxin systems with more than one component arose from the simpler ClyA PFT remains an open question. From direct comparison of toxin architectures (Figure 1a,b and Figure 2), it is apparent that the two-component YaxAB complex features modest, distinctly localized structural motions of its two subunits, which is in contrast to the major reorganization observed in ClyA.

## 5. Exposure of the Transmembrane Domains in ClyA-Like Toxins

**Figure 3:**
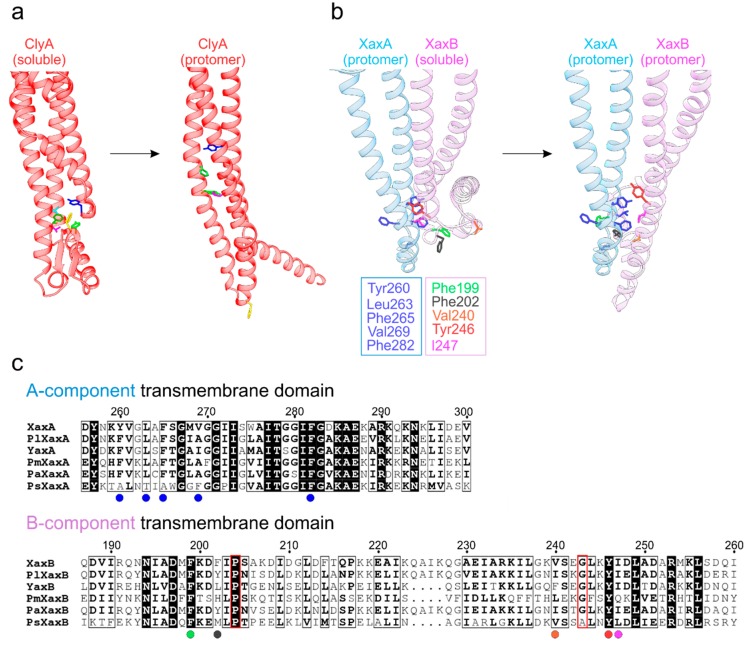
Differences in the lytic apparatus between one- and two-component ClyA-like toxins. (**a**) Exposure of the ClyA transmembrane domain by rearrangement of the hydrophobic core centered on F190 (colored gold). The remaining residues repack into the hydrophobic core of the protomer while F190 now projects into the membrane interior. (**b**) Exposure of XaxB’s (soluble: PDB ID 6GY7; pore: PDB ID 6GY6) lytic domain upon interaction with XaxA (soluble: PDB ID 6GY8), adapted from Schubert et al. [8]. The protomeric conformation of XaxB is stabilized by forming a tight interface with XaxA. (**c**) Sequence conservation of transmembrane domains in XaxA and XaxB orthologues. Residues highlighted in b) are indicated by colored circles below their positions. The hinge elements (P204 and G243) of the B-component are framed in red. Pl = (*Photorhabdus luminescens*), Pm = (*Proteus mirabilis*), Pa = (*Providencia alcalifaciens*), Ps = (*Pseudomonas syringae*).

α-PFTs from the ClyA family bear crucial membrane-active domains in the protein regions encompassing helices α4 to α5, which includes the toxin-specific structural building blocks located between them. For ClyA and XaxAB/YaxAB, structures are available both for the monomeric and protomer states, offering insights into the remarkable conformational transitions *en route* to membrane insertion (Figure 3). Intriguingly, in ClyA (Figure 3a) there is a hydrophobic cluster centered on phenylalanine 190 (as part of a structural element termed the ‘beta tongue’) in the soluble state, which suffers major rearrangements in the presence of a membrane (or an appropriate detergent in vitro) [9]. This module, together with helix α1, undergoes a substantial morph to form the α-helical transmembrane segment in the resulting protomeric ClyA. 

Recent X-ray and cryo-EM structures on the bimolecular XaxAB and YaxAB systems shed light on the lytic principles of these ClyA-related α-PFTs. Schubert et al. [8] proposed a plausible mechanism for the release of the XaxAB transmembrane domain by modeling a possible dimeric pre-pore form of XaxA and XaxB in protomeric and soluble conformations, respectively. Figure 3b illustrates the shift from this putative intermediate to the all-protomeric state. In the proposed model, Xa xA^protomer^ delivers a series of solvent-exposed hydrophobic side chains, decorating its foot domain (encompassing helices α4 and α5), in close proximity to hydrophobic residues of the XaxB^soluble^ counterpart (including the α4′ and α4′′ helices unique to the B component). Upon transformation to the all-protomeric XaxA-XaxB dimer, these residues now form a tight apolar interface. Furthermore, proline 204 and glycine 243 might act as hinge regions that facilitate the opening of the B-component foot domain [7,8]. Notably, the hydrophobic character of the residues in both the A and B subunits engaged in this transmembrane segment are conserved across human, insect and plant pathogens (Figure 3c), pointing to a unified lytic principle.

## 6. Assembly Pathways of ClyA-Like Pores

**Figure 4:**
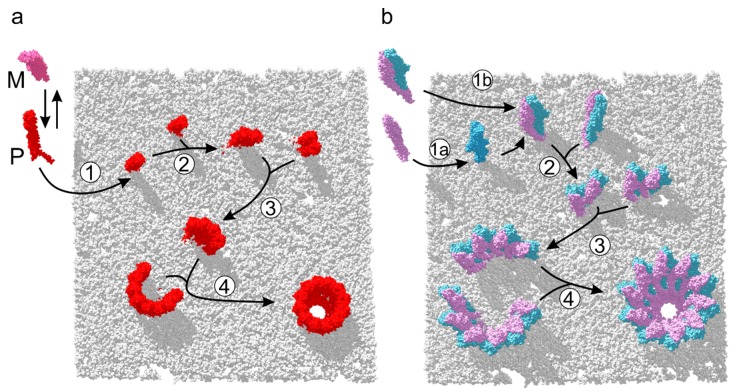
Assembly of one- and two-component ClyA-like PFTs. (**a**) Suggested pathway of ClyA pore formation [19]. In presence of a membrane, the ClyA monomer (M) adopts its protomeric conformation (P), and inserts into the membrane (1). Initial dimerization (2) leads to formation of multimers (3), which associate rapidly to closed dodecameric pores (4). (**b**) Proposed pathway of XaxAB-like assemblies, based on structural and biochemical data [7,8]. A-B dimers consecutively oligomerize on the target membrane (1a) or in solution (1b). Once the A-B dimer is membrane inserted (2), it associates with additional A-B units to form multimers (3) that assemble into closed pores (4).

Recently, the precise assembly pathway of the homooligomeric ClyA pore has been characterized by single-molecule spectroscopic studies [19]. These experiments have revealed that in the presence of target membranes or detergent, soluble ClyA evolves to its binding-competent protomeric conformation. Membrane-bound protomers proceed to form oligomers, which in turn associate rapidly with sterically compatible multimers, yielding closed dodecameric pores (Figure 4a). Structural and biochemical investigations of the orthologous XaxAB and YaxAB two-component toxins [7,8] have shed light on the diverging roles played by each subunit in the process of pore formation. The A-B dimer was identified to be quasi-protomeric, exhibiting a large functional interface and exposing contact sites to bind adjacent dimers. Although X-ray and cryo-EM studies have depicted the initial and final assembly states of the pore maturation process of XaxAB-like toxins, data about the dynamics of the process remains absent. Biochemical dissection of subunit membrane activity in vitro was performed on the YaxAB system [7], which supports a sequential mode of action on target membranes. In this scenario, YaxA binds first and recruits YaxB to initiate pore assembly. In contrast, data on XaxAB suggest that subunits dimerize first, followed by membrane insertion and further oligomerization [8]. Both routes are illustrated in Figure 4b, which eventually form protomeric, membrane-bound A-B dimers. Once heterodimer formation is completed, the pathway might proceed analogous to ClyA, with multimers of A-B dimers associating to form closed pore complexes [19].

## 7. Assembly of Three-Component Membrane Pores: Nhe and Hbl Toxins

Many years of research have established important roles in virulence for the *B. cereus* tripartite PFTs Nhe and Hbl [26,27,28,29,30,31]. These PFTs have been described as instrumental in conferring the diarrhea type of food poisoning. Progress on obtaining structural data on the pore assemblies of these complex toxins has been slow, while a body of biochemical investigations on Nhe in particular has substantiated several common features of their sophisticated mode of action.

The sequence identity of the individual subunits suggests a functional correspondence between NheA and Hbl-L2, NheB and Hbl-L1, as well as NheC and Hbl-B (Figure 1c). These striking similarities mirror current models on how each subunit contributes to the mechanism of pore formation. Indeed, pairwise binding experiments in solution could show stable complexes between NheB and NheC [32], as well as Hbl-B and Hbl-L1 [33]. Moreover, experiments have suggested that NheA and Hbl-L2 recruitment represent the final step in pore assembly for Nhe and Hbl toxins, respectively [16,34]. Interestingly, NheA itself cannot insert into membranes, whereas a subcomplex of NheBC forms permeable “pro-pores” in lipid bilayers [32]. These observations of consecutive association and divergent membrane-binding abilities recall the YaxAB system. In this simpler two-component regime, only YaxA is able to bind to membranes. Next, YaxB is recruited to initiate oligomerization of the lytic pore [7]. However, whether this sequential mechanism also holds in vivo for either two- or three-component toxins still remains to be clarified.

Intriguingly, biochemical analyses indicate a non-stoichiometric ratio between the three subunits of the Nhe pore [29]. Highest lytic activity was observed when the three components were present in a 10:10:1 stoichiometry (NheA:NheB:NheC). A study utilizing subunit and conformation-selective antibodies has provided first insights into the interaction between NheA and NheB in the context of the membrane-bound tripartite complex [35]. From a structural point of view, this suggests a 1:1 binding of NheA and NheB (akin to the YaxA-YaxB protomer), with a single NheC subunit eventually fulfilling a yet to be defined role. Consequently, molecular insights into these heterooligomeric supercomplexes are essential to reconcile biochemical investigations on these fascinating and multifaceted assemblies. Notably, the eukaryotic membrane attack complex (MAC), which adopts a spiral-shaped pore composed of 24 subunits, is so far the only other known PFT with asymmetric subunit composition [36]. MAC features one copy each of C5b, C6, C7, C8α, C8β, C8γ plus 18 copies of C9.

## 8. Conclusions

With the arrival of cryo-EM, more structures of ClyA-like pores will be reported in the near future. Amongst the established classes of PFTs, proteins of the ClyA-like family possess remarkable compositional variety. Nevertheless, since representative monomer X-ray structures are available for all known members of this extended toxin family, a common topological frame encompassing the 5HB is unambiguously confirmed. Therefore and as a result of divergent pore-forming mechanisms, encompassing homooligomeric, bipartite and tripartite systems, it can be expected that the membrane-active regions outside the common 5HB core motif will feature major structural distinctions (Figure 1a,b). Given the wide target spectrum of these toxins, including pathogens of humans, plants, insects and nematodes, α-PFTs will certainly be an important focus of structural infection biology in the coming years.
